# Fortification of Chinese Steamed Bread with *Glycyrrhiza* *uralensis* Polysaccharides and Evaluation of Its Quality and Performance Attributes

**DOI:** 10.3390/foods11152253

**Published:** 2022-07-28

**Authors:** Dongqi Guo, Xiuxiu Yin, Huan Cheng, Jianle Chen, Xingqian Ye

**Affiliations:** 1 National-Local Joint Engineering Laboratory of Intelligent Food Technology and Equipment, Zhejiang Key Laboratory for Agro-Food Processing, Ningbo Research Institute, College of Biosystems Engineering and Food Science, Zhejiang University, Hangzhou 310058, China; 15091893380@163.com (X.Y.); huancheng@zju.edu.cn (H.C.); chenjianle@zju.edu.cn (J.C.); 2 Production & Construction Group Key Laboratory of Special Agricultural Products Further Processing in Southern Xinjiang, College of Food Science and Engineering, Tarim University, Alar 843300, China

**Keywords:** Chinese steamed bread, quality, *Glycyrrhiza* polysaccharide, glycemic index, staling, sensory evaluation

## Abstract

Natural polysaccharides are new popular healthy food material, and the materials are widely used in various functional foods. The influences of polysaccharides from *Glycyrrhiza* *uralensis* on the quality and sensory properties of Chinese steamed bread (CSB), as well as the performance (starch digestion in vitro and starch staling) of CSB, were investigated in this study. The addition of *Glycyrrhiza* polysaccharide (GP) increased the specific volume of CSB in a dose-dependent manner, and the specific volume of CSB-2 was 2.55 mL/g. GP also contributed to the increase in hardness (from 1240.17 to 2539.34 g) and chewiness (893.85 to 1959.27 g) of fresh CSB. In addition, GP could maintain the integrity of the protein network within the CSB. The scores for sensory evaluation indicators of CSB-1 were relatively balanced. More importantly, the addition of GP altered starch digestive properties, and the content of the resistant starch (RS) was increased from 8.62 (CSB-0) to 43.46% (CSB-2). GP led to a significant reduction of the expected glycemic index (eGI) of CSB, and the eGI of CSB was decreased from 97.50 (CSB-0) to 73.8 (CSB-2), which was classified as a medium-GI (MGI) food. In addition, X-ray diffraction (XRD) and differential scanning calorimeter (DSC) revealed the addition of GP delayed the staling of CSB during storage. In general, adding the proper amount of GP could improve the quality of CSB and show the potential as a functional component of CSB to reduce the postprandial blood glucose level resulted by the CSB.

## 1. Introduction

In recent years, people’s interests in nutritional and healthy eating have increased. While meeting hunger and basic nutritional needs, foods also prevent diet-related diseases and improve the health of consumers [1,2]. Recently, a wide interest in the flour products industry has emerged, and many scholars have studied the replacement of ingredients in the recipes of flour products to improve the physicochemical-sensorial properties to fortify the functional of product. For example, Laganà et al. evaluated the effect of bergamot *Pastazzo* flour in shortbread biscuits [3], Giuffrè et al. studied the extra virgin olive oil on the physical–chemical–sensory properties of Italian cantuccini biscuits [4], and Azam et al. evaluated pomegranate seed powder as a functional ingredient in the cakes [5]. Chinese steamed bread (CSB) is one of the major traditional foods among Chinese and other Asian populations [6]. CSB is generally made from wheat flour, yeast/sourdough and water, which are fermented and then steamed [7]. However, wheat flour has lost a large number of dietary vitamins, fiber and other nutrients due to the development of the flour milling industry, thus heightening the risk of chronic diseases [8]. Recently, an increasing number of scholars have paid attention to how to improve the nutrition and consumer acceptance of CSB [9]. Therefore, the development of novel CSB with special tastes and enriched nutrition has become increasingly important [10]. In particular, CSB has contributed to the growth of health food markets in China, and the functional characteristics of CSB have been receiving increasing attention [11].

CSB is chiefly composed of refined wheat flour and tends to have a high glycemic index (GI > 90) [11,12]. High-glycemic index foods are related not only to diabetes but also to a variety of other chronic diseases, such as cardiovascular and disease obesity [13,14]. All types of functional components, such as red beetroot powder and purple sweet potato flour, have been fortified within the CSB formulation to improve nutrition and adjust the glycemic response of CSB [15,16]. Strategies to formulate CSB with decreased GI remain to be exploited to help patients with diabetes and other chronic diseases [11].

In addition, the production of CSB has become more industrialized and large scale with the rapid urbanization in recent years. To address this trend, the problem of CSB staling must be solved, which leads to the deterioration of quality during storage [17]. Specifically, the surface of CSB appears dry and cracked, and its structure becomes less compact and easy to drop, while the increased hardness and reduced elasticity and flavor during the storage period severely reduces consumer acceptance of CSB and brings enormous economic losses to producers [17,18]. One of the effective methods to inhibit the hardening and staling of CSB is to add food additives to CSB. The addition of natural polysaccharides (e.g., soluble dietary fiber, locust bean gum, konjac glucomannan) to CSB can soften it and delay staling, thus ensuring a longer shelf life [19].

Polysaccharides, natural polymeric carbohydrate molecules with unique functions, are widely distributed in organisms including plants, animals and microorganisms. Polysaccharides have been utilized as natural ingredients to design functional products in the cosmetics, agriculture, health food and pharmaceutical industries. Currently, a growing amount of research shows that polysaccharides have various biological activities, such as antioxidant, antidiabetic, prebiotic, anti-inflammatory, immunoregulation, hypolipidemic and hypoglycemic effects and antitumor properties [20]. Polysaccharides, such as pectin, xanthan and oat β-glucan, can not only improve specific volume, texture and crumb porosity and increase moisture retention in wheat flour products and decrease its staling rate during storage, but can also be used to create functional foods that can meet consumer needs for well-being [21]. *Glycyrrhiza* belongs to the Fabaceae family and has been highly valued as a medicinal plant and food worldwide for thousands of years. *Glycyrrhiza* polysaccharide (GP), as one of the main bioactive components of *Glycyrrhiza*, has received increasing attention. In this study, *Glycyrrhiza* polysaccharide was obtained from our laboratory. This is the first application of GP in CSB and thus unlike the other studies. As a natural polysaccharide, GP may improve the performance of CSB and provide health benefits that are mainly relate to its variety of biological activities [22,23]. Therefore, the CSB is good candidate to be incorporated with GP.

Therefore, the aims of the study were as follows: (i) to investigate the influences of adding GPs on the quality, sensory properties and the starch digestion of the CSB; and (ii) to evaluate its starch staling compared with that in the control sample during storage.

## 2. Materials and Methods

### 2.1. Materials

Multipurpose wheat core wheat flour (Mingmai 16, Species: *Triticum aestivum*, Genus: *Dinkel*, Jiangsu Tomorrow Seed Technology Co., Ltd., Nanjing, China; fat, 1.6 g/100 g; protein, 11.0 g/100 g; carbohydrate, 73.5 g/100 g, GB/T 1355, Beijing, China) was procured from Yihai Jiali Golden arowana Grain, Oil and Food Co., Ltd. (Shanghai, China). Instant dry yeast (*Saccharomyces cerevisiae*) was supplied by Angel Yeast Co., Ltd. (Yichang, Hubei, China). *Glycyrrhiza* polysaccharides were obtained from *Glycyrrhiza uralensis* Fisch with hot water extraction and GP was comprised 58.7% D-glucose, 19.71% D-galactose, 8.86% D-mannose, 8.64% L-arabinose, 1.82% D-galacturonic acid, 1.54% L-rhamnose, 0.52% L-fucose and 0.21% D-glucuronic acid (Dionex system) and had a molecular weight of 261.2 KDa (high-performance size exclusion chromatography).

### 2.2. Preparation of CSB

CSB was prepared as described by Cui with some modifications [16]. Briefly, freeze-dried powder of GP was added to wheat flour to reach proportions of 0% (control), 0.5%, 1% and 2% (*w*/*w*) in the flour mixture (200 g). Instant dry yeast (2 g) was mixed with 50 mL of warm pure water with stirring (50 rpm) for 5 min. Next, total flour and an appropriate volume of pure water were added to the yeast solution and kneaded until a smooth dough formed. The dough was fermented at 30 °C and 80–90% relative humidity (RH) for 120 min in a fermentation cabinet (RMF-18PC, Guangzhou Rongmai Baking Food Machinery Manufacturing Co., Ltd., Guangzhou, China). After fermentation, the dough was divided into appropriate pieces. Each piece was formed into a round shape by hand and fermented at 30 °C with 80–90% RH for 35 min in the same ferment cabinet. The proofed dough was steamed for 30 min using a stainless-steel steamer. The CSB was cooled for 1–2 h at room temperature before analysis. The test groups were named CSB-0 (0%), CSB-0.5 (5%), CSB-1 (1%) and CSB-2 (2%) according to their GP content.

### 2.3. Appearance and Internal Texture of CSB

Photographs of the appearance and cross-section of the CSB were taken by a camera (Canon 600D, Canon (China) Co. Ltd., Beijing, China). The Jmage J V1.8.0 software (National Institutes of Health, Bethesda, Maryland, USA)was used to analyze the digital image of stomata in cross-sections of CSB.

### 2.4. Specific Volume, Moisture Content and pH Measurement

The volume of CSB was measured by the millet displacement method, and the specific volume (SV) was calculated as the ratio of volume to the weight of CSB (mL/g). The moisture content and pH of each sample were measured according to the method recommended by Chinese National Standards (GB 5009.3-2016 and GB 21118-2007, respectively).

### 2.5. Textural Property Analysis (TPA)

A texture Analyzer (Universal TA, Shanghai TengBa Instrument Technology Co., Ltd., Shanghai, China) was used to analyze the textural properties of the CSB. The CSB was sliced into 2 cm × 1 cm × 1 cm cubes before the test. Samples were compressed to 50% of their original height utilizing a 35-mm piston probe (P/35). The extrusion tests were executed in start test mode at a pre-test and post-test speed of 1.00 mm/s to 5 mm at a power of 5.0 g.

### 2.6. Color of CSB

The color values of CSB (crust and crumbs) were determined by a colorimeter (ColorFlex, EZ, HunterLab. Inc., Reston, Virginia, USA). Differences in color were evaluated on the basis of CIE L*, a*, b* [24,25]. Three different spots on the CSB were randomly selected and measured. The whiteness index (WI) of CSB was calculated by the Hunter whiteness formula:

(1) $$\mathrm{WI}=100-\sqrt{{\left(100-L^{*}\right)}^{2}+a^{*}{}^{2}+b^{*2}}$$

### 2.7. CLSM of CSB

The microstructure of CSB samples was observed utilizing confocal laser scanning microscopy (CLSM, Leica, Wetzlar, Germany). The method was described by Tang with modifications [21]. In brief, CSB was cut into approximately 1 mm thick slices using a sharp blade and placed on microscope slides. The proteins and starches in CSB were labeled with fluorescein isothiocyanate (FITC, 0.05%, *w*/*v*) and rhodamine B (0.05%, *w*/*g*) for 2–3 min, washed with deionized water and then covered with coverslips. The excitation wavelengths for red and green labels were 488 and 543 nm, respectively. Samples from every experiment were examined in four or five areas, and each representative image was obtained.

### 2.8. Starch Digestion In Vitro and Expected Glycemic Index (eGI)

The in vitro starch digestibility of CSB was determined utilizing the method of Zhu et al. and Englyst et al. with several modifications [15,26]. Briefly, a piece of the CSB (1 g) was added into 2% slurry (sodium acetate buffer, pH 5.2) before mixing with pancreatic amylase, amyloglucosidase and invertase for hydrolysis for 2 h at 37 °C. After 0, 20, 40, 60, 90, 120 and 180 min, aliquots (0.5 mL) were taken and then added into 2.5 mL of anhydrous ethanol to terminate the reaction. The amount of glucose from hydrolysis was quantified utilizing GOPOD reagent. According to Englyst’s classification of starches, rapidly digestible starch (RDS), slowly digestible starch (SDS) and resistant starch (RS) were calculated in accordance with the following formulas:

RDS = 0.9 × G20; SDS = 0.9 × (G120 − G20); RS = 0.9 × (TS − G120).

where 0.9 is the conversion coefficient of the relationship between glucose and starch quality; G20 and G120 are the glucose content (g) in the samples after 20 min and 120 min, respectively; and TS is the total starch content (g), which was analyzed utilizing the method described by the manufacturer of the Total Starch Assay Kit (K-TSTA 04/2009; Shanghai Xinrui Biotechnology Co., Ltd., Shanghai, China).

The enzymatic hydrolysis of CSB samples was analyzed according to the method of Goñi et al. [27]. Briefly, the starch hydrolysis curve was used to obtain the area under the curve (AUC) for calculation by dividing the graph into trapezoids. White bread (BreadTalk, Wal-Mart, Hangzhou, China) was used as a standard sample with a hydrolysis index (HI) of 100%. HI = AUC_sample_/AUC_reference_. The eGI of CSB was calculated according to the equation: eGI = 39.71 + 0.549 × HI [27,28].

### 2.9. Sensory Evaluation of CSB

The sensory evaluation was performed according to Chinese GB/T 35991-2018. A discriminatory sensory test of the fresh CSB was performed in 14 people (7 females and 7 males, aged of 22–28 years) who consumed CSB as their staple food and were not smokers. CSB was provided to 14 training panelists within 1 h of steaming. Eight criteria were evaluated, including specific volume, color, springiness, appearance, structure, stickiness, toughness and flavor, which contributed to 25%, 10%, 10%, 10%, 20%, 10%, 10% and 5% of the total score, respectively (Table 1).

### 2.10. Determination of Recrystallization of Stored CSB

After cooling at room temperature for 1 h, fresh CSB was kept in storage bags and stored for 5 days at 4 °C for analysis. CSB samples for X-ray measurement were vacuum freeze-dried and ground into a powder and then passed through a 100-mesh sieve. Samples were analyzed on a Bruker D8 Advance diffractometer (X’Pert 3 Powder, Malvern PANalytical, Almelo, The Netherlands). Diffraction patterns were recorded at a 2θ angle from 4° to 40° with the voltage and current set to 40 kV and 40 mA, respectively, using Cu Kα radiation (λ = 0.15406 nm). The Jade 7.0 Software (MDI Materials Date Inc., Livermore, California, USA) was used for spectral analysis.

### 2.11. Determination of the Regenerative Heat Enthalpy of Stored CSB

The thermal properties of CBS at 4 °C for 5 days were analyzed using a differential scanning calorimeter (DSC, Mettler Toledo, Zurich, Switzerland) with high-purity nitrogen (N_2_) gas purge. CSB samples were obtained according to Section 2.10. The instrument was calibrated utilizing indium as a standard, and an empty aluminum pan was used as a reference. Samples (3-3.2 mg) were weighed into an aluminum pan (10 μL), triple the volume of deionized water was added and the samples were hermetically sealed and then equilibrated overnight at 4 °C. The sample pan was heated from 30 to 110 °C at 10 °C/min. Retrogradation enthalpy (ΔH) was expressed in J/g sample (on a dry matter basis) [29].

### 2.12. Statistical Analysis

Statistical analyses were performed with SPSS 21 (IBM Corp., New York, NY, USA). One-way analysis of variance (ANOVA) with Duncan’s test was used to determine significant differences between means (*p* < 0.05).

## 3. Results and Discussion

### 3.1. Appearance and Crumb Structure of CSB

**Figure 1:**
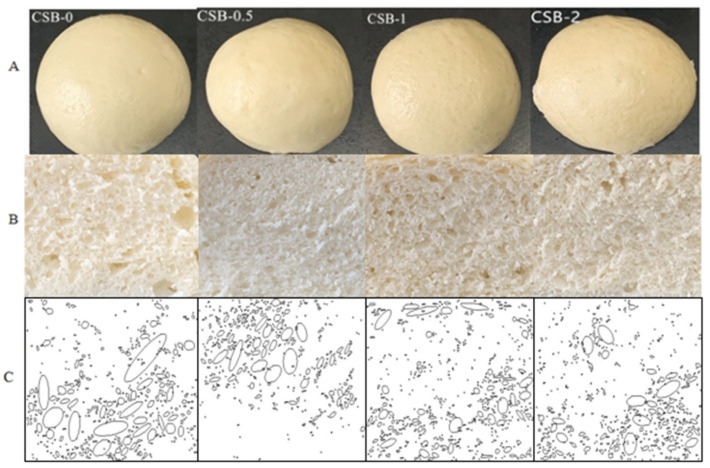
Appearances (**A**), cross-sections (**B**) and Fourier fitting stomata image (**C**) of fresh CSB.

The surface views, cross-sections and Fourier fitting stomata image (Dpi 350 × 285) of CSB are shown in Figure 1A–C. In general, the addition of GP had no significant affect on the color of crusts and obviously changed the crumb structure, as indicated by distinct differences in the crumb structure (size and number of gas cells). With the increase of GP addition, the stomatal number and area fraction of CSB increased from 402 to 553, while the area fraction increased from 10.80% to 17.71%. In addition, the stomatal area of CSB-0 was from 2620 to 1 μm^2^, the stomatal sizes were significantly different and the distribution was not uniform. Compared with the control sample, CSB with GP had a more uniform and finer particle structure, suggesting that the addition of GP could improve the CSB crumb structure. The similar results were observed when exopolysaccharides added to the Chinese steamed bread [21].

### 3.2. Specific Volume, Moisture Content and pH of CSB

The incorporation of GP increased the specific volume of CSB in a dose-dependent manner (Table 2). For example, the substitution level of GP was 2%, and the specific volume of CSB-2 was 2.55 mL/g, which was higher than that of CSB-0 (control) by 10.39%. The volume of CSB was positively correlated with the gas holding capacity. The increases could be due to the phenomenon, whereby GP can strengthen the gluten network of CSB, increasing the gas holding capacity of CSB and the mechanical strength of dough during proofing and steaming. Similar alterations in specific volume have been reported in CSB fortified with other functional polymers such as medium-viscosity konjac glucomannan and high-viscosity konjac glucomannan [30]. Moisture content plays a crucial role in CSB, especially during storage. The addition of GP had a slight influence on the moisture content (39.48-42.57%) of fresh CSB (Table 2). This may be mainly attributed to the steaming process which fully hydrated the CSB ingredients. The moisture contents of CSB in this study were similar to those of CSB in previous studies [31]. The addition of GP did not significantly affect the pH of CSB and generally remained constant at 5.60 (Table 1). The pH values of each sample conformed to Chinese National Standards (GB 21118-2007).

### 3.3. Textural Properties of CSB

The texture of food chiefly refers to its organizational properties, which are associated with the sensory and edible properties of food. TPA is an objective sensory analysis method that consists of compressing a bite-size piece of food twice in a reciprocating motion that simulates jaw movement and provides values of parameters such as hardness, springiness, cohesiveness and chewiness [32]. The textural properties of CSB are shown in Table 2. Generally, GP had varied influences on the textural properties of CSB. The addition of GPs contributed to the increase in hardness (from 1240.17 to 2539.34 g) and chewiness (893.85 to 1959.27 g) of fresh CSB, while the cohesiveness and springiness changed slightly. However, this relationship was not linear with the concentration of GP. The CSB with GP addition had the higher hardness than that of the control, which might be attributed to that it had denser crumb and more compact gas cells, thereby increasing the hardness. However, it was not consistent with the results of specific volume. This was different from other scholars’ results [15,33]. Another reason might be that the increased hardness could be caused by the interaction between GP and wheat gluten, which promoted the formation of a gluten network. Chewiness refers to the energy required to break food down into smaller pieces by mastication [13]. The increased chewiness reflects the compact structure of CSB with the addition of GP, which requires more energy and longer oral processing before swallowing. Cohesiveness is indicative of the strength of internal bonds making up the CBS crumb [34]. The cohesiveness of CSB-1 (2.50 ± 0.73) and CSB-2 (2.12 ± 0.22) were higher than that of the control. This may reflect that the microstructure of CSB was much reinforced due to the gluten/starch matrix tightness by GP. The altered texture of CSB induced by GP addition (e.g., increased hardness) may not be a disadvantage for consumer acceptance. This is because there is a great diversity in consumer preference of the CSB texture in China [6].

### 3.4. Color of CSB

The addition of GPs observably affected the color of both the crust and crumbs of the CSB (Table 3). GP addition reduced the L* (from 35.38 to 29.71) and WI (34.93 to 29.33) of the CSB crust. Similar to the influence on crumb color, the addition of GP decreased the L* and WI in a concentration-dependent manner. It suggested that the addition of GP reduced the brightness and overall whiteness of CSB. This was attributed to color of GP was darker than that of wheat flour. The color of CSB was not only depended on color of the materials, but also on the change of internal microstructure and composition [17]. In the CSB, a* and b* were not linearly related to the content of GP. This may be due to the uneven distribution of GP and the selection of random spots on the CSB for analysis. The changes in color are not necessarily a negative factor related to consumer acceptance for the purpose of new product introduction [35].

### 3.5. Effects of GP on the Microstructure of the Protein Network of CSB

**Figure 2:**
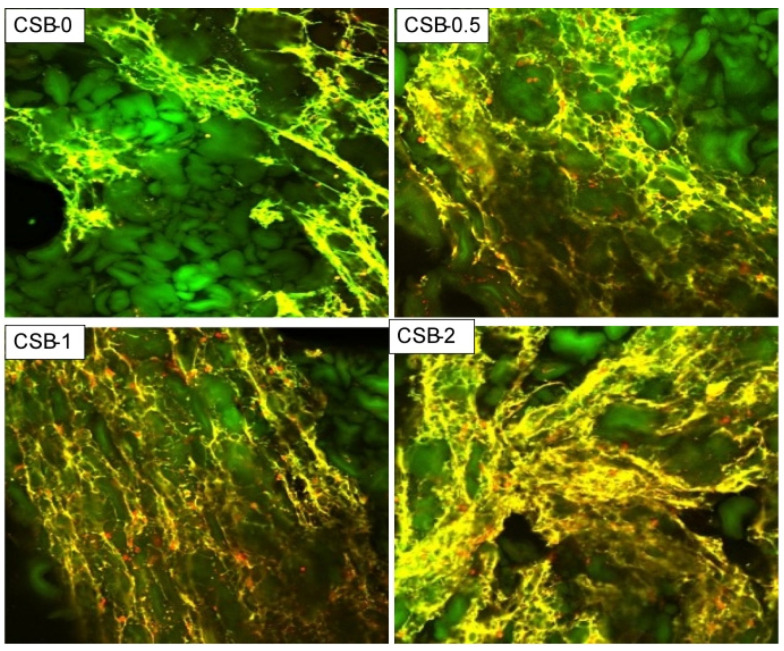
CLSM micrographs of fresh CSB.

The effects of GP on the microstructure of the protein network within the CSB were observed by CLSM. The CLSM images indicated that the microstructure of the protein network (yellow) within the CSB prepared with GP became denser and more homogeneously distributed surrounding the starch granules (green) than the control sample, roughly in a concentration-dependent manner (Figure 2). This confirmed that the addition of GP resulted in a cross-linking reaction with the gluten protein through hydrogen bonding, hydrophobic interactions or steric entanglement of polymer chains, which induced the polymerization of gluten protein [1,36]. This result was in accordance with a previous study showing that GP had a high molecular mass and rich branched structure [22]. Adding GP could maintain the integrity of the protein network within the CSB. A continuous and uniform protein network is the foundation of CSB texture and can effectively prevent CSB dehydration and shrinkage.

### 3.6. In Vitro Starch Digestion and eGI

The consumption of foods high in starches is closely associated with diet-related chronic metabolic diseases, including obesity, diabetes and hypertension. The main ingredient of CSB is starch. Generally, excessive intake of RDS rapidly increases postprandial blood glucose (PBG), while SDS is helpful for balancing PBG, and RS is beneficial for improving intestinal health and preventing colon cancer [37]. As shown in Table 4, the types of starch components in the CSB were remarkably influenced by GP addition. The RDS and SDS in the CSB were decreased by GP addition, while RS were significantly improved. For instance, the contents of the RDS and SDS were decreased from 40.01% and 3.80% in the CSB-0 (control) to 16.12% and 40.43% in the CSB-2, respectively. The content of RS was increased from 8.62 (CSB-0) to 43.46% (CSB-2). However, the RDS, SDS and RS were not linearly related to the content of GPs in CSB. The addition of GPs to CSB significantly altered the eGI (Table 3). The eGI of CSB-0 was 97.50, which was similar to result of Jiao et al. [38]. The glycemic index (GI) of CSB indicated that it was a high-GI (≥98) food. Other studies also demonstrated that CSB had a higher GI. The addition of GPs could reduce the eGI of the CSB. For instance, the eGI of CSB was decreased from 97.50 (CSB-0) to 73.8 (CSB-2), which was classified as a medium-GI (MGI) food.

A series of factors contributed to the reduced starch digestibility in vitro and eGI. Polysaccharides can affect starch digestion by changing its physical and chemical properties. In addition, polysaccharides can prevent starch grains from being hydrolyzed by amylase by encapsulating them, and thus affecting the release of free glucose and resulting in a reduced glycemic response [39]. The decreased glycemic load of CSB fortified with GP is beneficial for consumers with obese and diabetic conditions. Therefore, it may have great potential as a functional food [40]. The results indicated that GP could inhibit the digestion in vitro and hydrolysis of starch to some extent, thereby decreasing the starch hydrolysis rate and eGI value of CSB.

### 3.7. Sensory Evaluation of CSB

**Figure 3:**
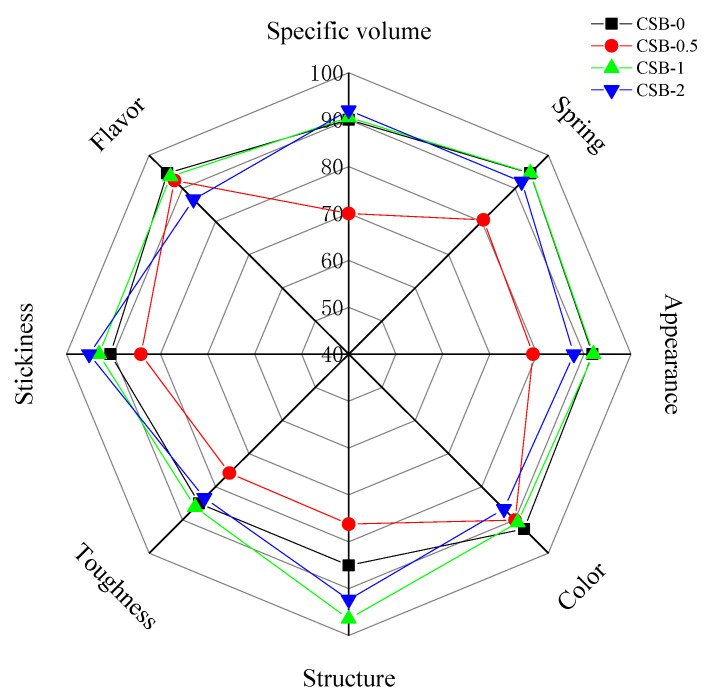
Sensory scores of quality attributes of CSB.

The addition of GP obviously changed the sensory quality of CSB to varying degrees (Figure 3). The GP addition tended to roughly decrease the appearance score of CSB. This is largely due to the increased surface roughness. This was in accordance with the results obtained from appearance (Section 3.1). The color of both crust and crumb were lower whiteness than that of the control. This agreed with the results with the color-meter as shown in Section 3.4. The score of specific volume and structure accorded with the results of Section 3.1 and Section 3.2, respectively. The addition of GP increased the hardness of CSB while decreasing the stickiness and springiness. The toughness of CSB was slightly affected by GP addition, although TPA analysis of CSB indicated that the addition of GP addition increased the chewiness of CSB (Table 2). In this regard, the lack of correlation between sensory and instrumental results may indicate that definitions of the two terms with the same nomenclature have fundamental differences [15]. On the other side, the results showed that the addition of GP was decreased the flavor score of CSB at levels 2%, which may be unacceptable for some people due to the poor flavor caused by higher concentrations of GP. Generally, the scores for sensory evaluation indicators of CSB-1 were relatively balanced. CSB had more desirable functional activity, as shown in Section 3.6, with increasing levels of GP. Therefore, strategies to improve the edible quality of CSB remain to be investigated. Moreover, further analysis of the sensory evaluation of CSB can take advantage of the method of Bayat et al. [41].

### 3.8. Staling of Starch in CSB

#### 3.8.1. XRD Analysis

**Figure 4:**
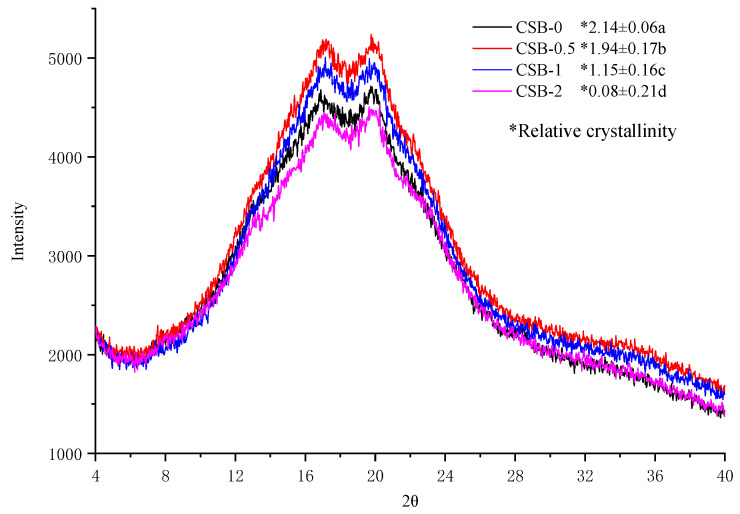
XRD pattern of CSB after storage for 5 days at 4 °C.

Staling of CSB is generally due to the retrogradation of starch, which usually occurs together with the development of a crystalline matrix [42]. To examine the influence of GP on the crystal structure of CSB after storage for 5 days at 4 °C, X-ray diffraction analysis was performed. The diffractograms and relative crystallinity are shown in Figure 4. Two obvious peaks at 17° and 20° were observed form the XRD spectra of CSB, which were characteristic recrystallization peaks of amylose and amylopectin. The result was similar to a previous study declaring that konjac glucomannan was added to steamed bread [30]. The X-ray diffraction patterns of all CSB were similar, but the relative crystallization obviously decreased with increasing GP content. The results indicated that GP can retard the retrogradation of starch in CSB, thereby delaying the staling of CSB during the storage to a certain extent.

#### 3.8.2. DSC Analysis

Starch retrogradation has long been used as an indicator to assess the extent of staling as it is the primary factor leading to the staling of starch-based products. The long-term development of crystallinity in starches is due to the amylopectin fraction, which is involved in the staling process [43]. As shown in Table 4**,** the onset (*T*o), peak (*T*p) and conclusion (*T*c) temperatures of the CSB samples were similar. For example, the *T*p values of CSB-0, CSB-0.5, CSB-1 and CSB-2 were 54.31, 54.39, 54.34 and 54.94 °C, respectively. The melting enthalpy of retrograded amylopectin (an endothermic peak at approximately 40 °C to 70 °C) was found in all CSB samples, indicating the formation of amylopectin crystals after storage for 5 days at 4 °C, which was consistent with the results of Sha et al. [44]. The results also demonstrated a linear relationship between the decrease in melting enthalpy and GP content. The value of retrogradation enthalpy of CSB-2 was 2.85 J/g, which was lower than that of CSB-0 (3.76 J/g). This was due to the enhancement of the fluidity of amylopectin molecules by polysaccharides through their interference with the rearrangement process of amylopectin molecules and, thus, reduction in the degree of amylopectin regeneration. Therefore, GP may be used as an effective additive in CSB to delay the staling of CSB while maintaining a better quality of CSB.

## 4. Conclusions

The results obtained in this study indicated that GP had notable influences on the quality, starch digestion and staling properties of CSB. The addition of GP had a positive effect on the moisture content and specific volume of CSB. The hardness, chewiness and gumminess of CSB increased with the addition of GP, while the cohesiveness, springiness and resilience changed slightly. The CSB appeared as a more conspicuous yellow crust with increasing GP concentration. GP could improve the CSB crumb structure by maintaining the integrity of the protein network in CSB. The scores for sensory evaluation indicators of CSB-1 were relatively balanced among the CSB samples with GP addition. The contents of RDS and SDS in CSB were decreased by GP addition, whereas that of RS was increased while reducing eGI of CSB. Moreover, GP could effectively inhibit wheat starch recrystallization, thereby delaying the staling process of CSB to a certain extent. The report may stimulate the application of natural polysaccharides for novel functional food production and development. Furthermore, the functional evaluation of CSB with polysaccharides should be carried out.

## Figures and Tables

**Table 1:** The criterion of sensory evaluation of CSB.

Characteristics	Criterion of Evaluation
Specific volume (25)	When the specific volume is 2.8 mL/g or more, a full score of 25 is given; a minimum score of 10 is given for a specific volume of 1.8 mL/g or less; for every 0.1 drop in volume between 2.8 and 1.8 mL/g, 1.5 scores will be deducted.
Stickiness (10)	Finger press resilience is good: 8–10 scores; finger press resilience is weak: 6–7 scores; Finger press is not rebound or press difficult: 4–5 scores.
Color (10)	Good gloss: 8–10 scores; slightly dark: 6–7 scores; gray: 4–5 scores
Appearance (10)	Smooth surface: 8–10 scores; shrinkage, collapse, bubble, hard or hot spots (brown spots on the skin): 4–7 scores
Structure (20)	Pores fine and uniform: 18–20 scores; pores are fine and uniform, with individual bubbles: 13–17 scores (when edge and epidermis have separation phenomenon, 1 score will be deducted); pores are basically uniform, but with one of the following conditions (too close, slightly more bubbles or pores uniform but slightly rough structure): 10–12 scores; uneven stomata or rough structure: 5–9 scores
Toughness (10)	Toughness is strong: 8–10 scores; toughness is general: 6–7 scores; toughness is poor, dross or chewing dry: 4–5 scores
Stickiness (10)	Refreshing and not sticky teeth: 8–10 scores; slightly sticky: 6–7 scores; chewing is unpleasant and very sticky: 4–5 scores;
Flavor (5)	Natural aroma of normal wheat: 5 scores; tasteless: 3–4 scores; smell: 2–3 scores.

**Table 2:** Specific volume, moisture content, pH and textural properties of CSB.

Sample	Specific Volume (mL/g)	Moisture Content (%)	pH	Hardness (g)	Springiness	Chewiness (g)	Cohesiveness
CSB-0	2.31 ± 0.21 a	39.48 ± 1.47 a	5.40 ± 0.02 a	1240.17 ± 94.68 a	0.94 ± 0.01 a	893.85 ± 62.03 a	1.63 ± 0.30 a
CSB-0.5	2.36 ± 0.03 ab	40.82 ± 0.84 ab	5.52 ± 0.03 a	2539.34 ± 25.00 b	0.90 ± 0.03 a	1959.27 ± 70.59 b	1.41 ± 0.23 b
CSB-1	2.47 ± 0.30 c	41.84 ± 0.67 bc	5.61 ± 0.04 a	1338.18 ± 18.67 c	0.92 ± 0.01 a	1083.96 ± 18.58 c	2.50 ± 0.73 c
CSB-2	2.55 ± 020 cd	42.57 ± 0.74 cd	5.71 ± 0.03 a	1858.91 ± 27.12 d	0.91 ± 0.03 a	1410.57 ± 59.17 d	2.12 ± 0.22 d

Means ± standard deviation (*n* = 3). Values in in the same column with different letters differ significantly (*p* < 0.05).

**Table 3:** Color of CSB.

Sample	Crust				Crumb			
	*L* *	a*	b*	WI	L*	a*	b*	WI
CSB-0	35.38 ± 0.94 a	−1.03 ± 0.03 a	7.59 ± 0.18 a	34.93 ± 1.00 a	43.91 ± 3.27 a	−1.05 ± 0.28 a	12.72 ± 1.70 a	42.48 ± 2.80 a
SCB-0.5	33.78 ± 1.82 b	−1.09 ± 0.02 ab	5.81 ± 0.13 b	33.52 ± 1.72 b	41.39 ± 1.11 b	0.01 ± 0.20 b	14.50 ± 0.68 b	39.62 ± 1.15 b
CSB-1	33.66 ± 1.10 bc	−1.16 ± 0.03 c	5.57 ± 0.22 c	33.42 ± 1.20 bc	40.00 ± 0.35 c	−0.65 ± 0.09 c	13.39 ± 0.23 c	38.52 ± 0.30 c
CSB-2	29.71 ± 0.30 d	−0.84 ± 0.04 d	7.23 ± 0.24 ad	29.33 ± 0.35 d	39.00 ± 1.27 d	−0.74 ± 0.10 d	11.83 ± 0.39 d	37.86 ± 1.50 d

Means ± standard deviation (*n* = 3). Values in in the same column with different letters differ significantly (*p* < 0.05).

**Table 4:** Starch nutritional fractions, eGI and DSC parameters of CSB.

Sample	RDS (%)	SDS (%)	RS (%)	eGI	*T*o (℃)	*T*p (℃)	*T*c (℃)	Δ*H* (J/g)
CSB-0	40.01 ± 7.93 a	53.80 ± 3.75 a	8.62 ± 2.95 a	97.50 ± 3.05 a	47.03 ± 0.65	54.31 ± 0.22	62.34 ± 2.10	3.76 ± 0.13 a
CSB-0.5	20.82 ± 2.84 b	42.80 ± 9.36 b	36.38 ± 1.18 b	82.99 ± 4.15 b	46.17 ± 1.05	54.39 ± 0.17	64.35 ± 1.79	3.57 ± 0.02 b
CSB-1	24.05 ± 0.71 c	51.19 ± 4.17 c	24.76 ± 3.89 c	88.69 ± 1.50 c	45.55 ± 1.69	54.34 ± 0.03	65.76 ± 0.60	3.44 ± 0.18 c
CSB-2	16.12 ± 0.43 d	40.43 ± 0.00 d	43.46 ± 0.43 d	73.38 ± 0.95 d	46.12 ± 0.80	54.94 ± 0.23	64.57 ± 0.66	2.85 ± 0.21 d

Means ± standard deviation (*n* = 3). Values in in the same column with different letters differ significantly (*p* < 0.05).

## Data Availability

The data presented in this study are available on request from the corresponding author.
